# Supplementary material for: STREAMING-tag system reveals spatiotemporal relationships between transcriptional regulatory factors and transcriptional activity
Source: Nat Commun. 2022 Dec 20;13:7672. doi: 10.1038/s41467-022-35286-2 (PMC9768169; doi:10.1038/s41467-022-35286-2)
Supplement: Supplementary file 1 — Supplementary Information [file 41467_2022_35286_MOESM1_ESM.pdf]

Supplementary figures and legends

a

```
35.1% identity in 251 residues overlap; Score: 367.0; Gap frequency: 2.8%

NeoR-I            12 LYGYKWARDNVGQSGATIYRLYGKPDAPELFLKHGKGSVANDVTDEMVRLNWL-TEFMPL
NeoR-II           25 LFGYDWAQQTIGCSDAAVFRLSAQ-GRPVLFVKTDLSGALNELQDEAARLSWLATTGVPC
                     * ** **    * * *   **      * ** *       *   **  ** ** *   *

NeoR-I            71 PTIKHFIRTPDDAWLLTTAIPGKTAFQVLEEYPDSGENIVDALAVFLRRLHSIPVCNCPF
NeoR-II           84 AAVLDVVTEAGRDWLLLGEVPGQ---DLLSSHLAPAEK-VSIMADAMRRLHTLDPATCPF
                                  ***    **      *       *  *   *   ****      ***

NeoR-I           131 NSDRVFRLAQAQSRMNNGLVDASDFDDERNGWPVEQVWKEMHKLLPFSPDSVVTHGDFSL
NeoR-II          140 DHQAKHRIERARTRMEAGLVDQDDLDEEHQGLAPAELFARLKARMPDGDDLVVTHGDACL
                           *   *  **  ****  * * *  *              *   * ******  *

NeoR-I           191 DNLIFDEGKLIGCIDVGRVGIADRYQDLAILWNCLGE-FSPSLQKRLFQKYGIDNPDMNK
NeoR-II          200 PNIMVENGRFSGFIDCGRLGVADRYQDIALATRDIAEELGGEWADRFLVLYGIAAPDSQR
                      *     *   * ** ** * ****** *       *        *    ***  **

NeoR-I           250 LQFHLMLDEFF
NeoR-II          260 IAFYRLLDEFF
                       *   *****
```

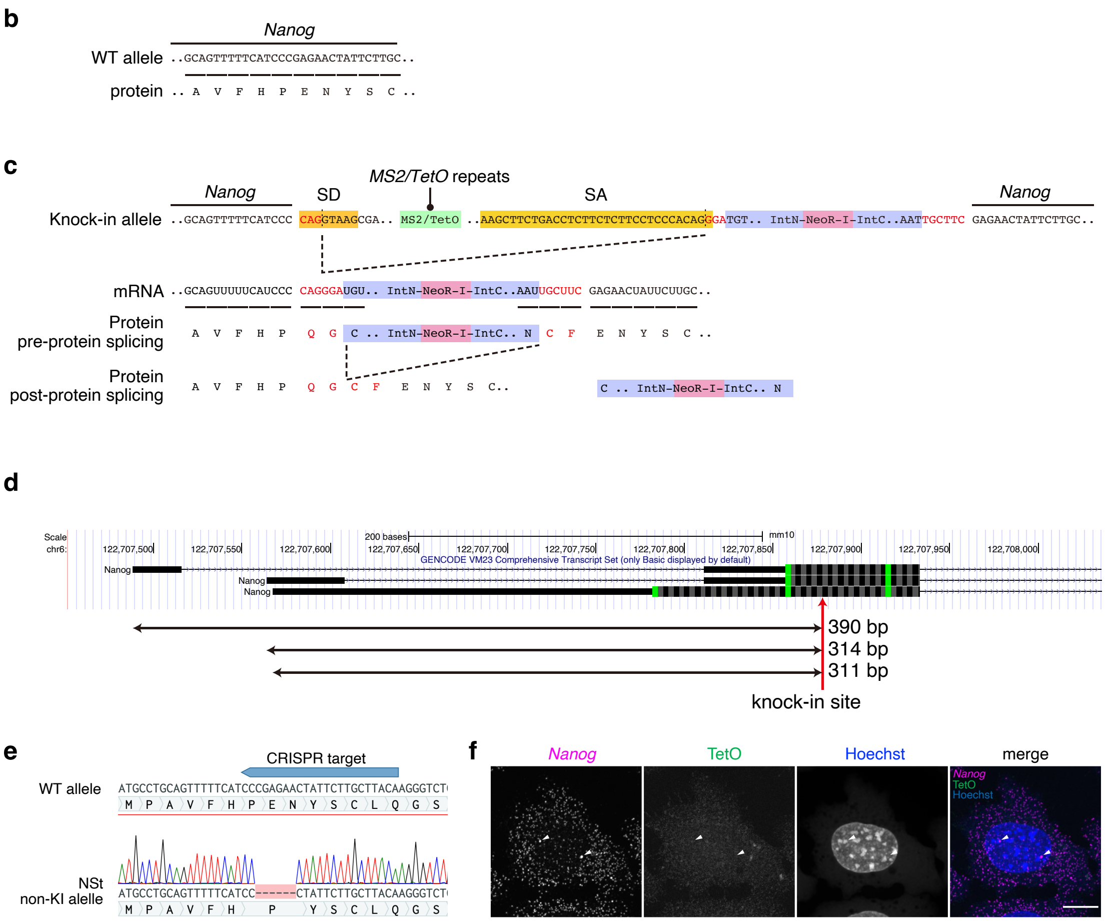

Supplementary Fig. 1 Knock-in of STREAMING-tag to *Nanog* and its effects. a, Comparison of amino acid sequences of NeoR-I and NeoR-II. **b**, Genomic DNA and coding amino acid sequences of wild-type (WT) around the STREAMING-tag knock-in site in *Nanog*. **c**, Effect of STREAMING-tag knock-in into *Nanog* on gene products. **d**,

STREAMING-tag knock-in site in *Nanog* and distances from transcription start sites to the knock-in site. **e**, DNA sequence of the WT allele in NSt cells, in which a deletion of six base pairs was introduced. **f**, Single-molecule fluorescence *in situ* hybridization (smFISH) analysis of WT cells. Arrowheads indicate *Nanog* transcriptional spots. Scale bar, 10 μm.

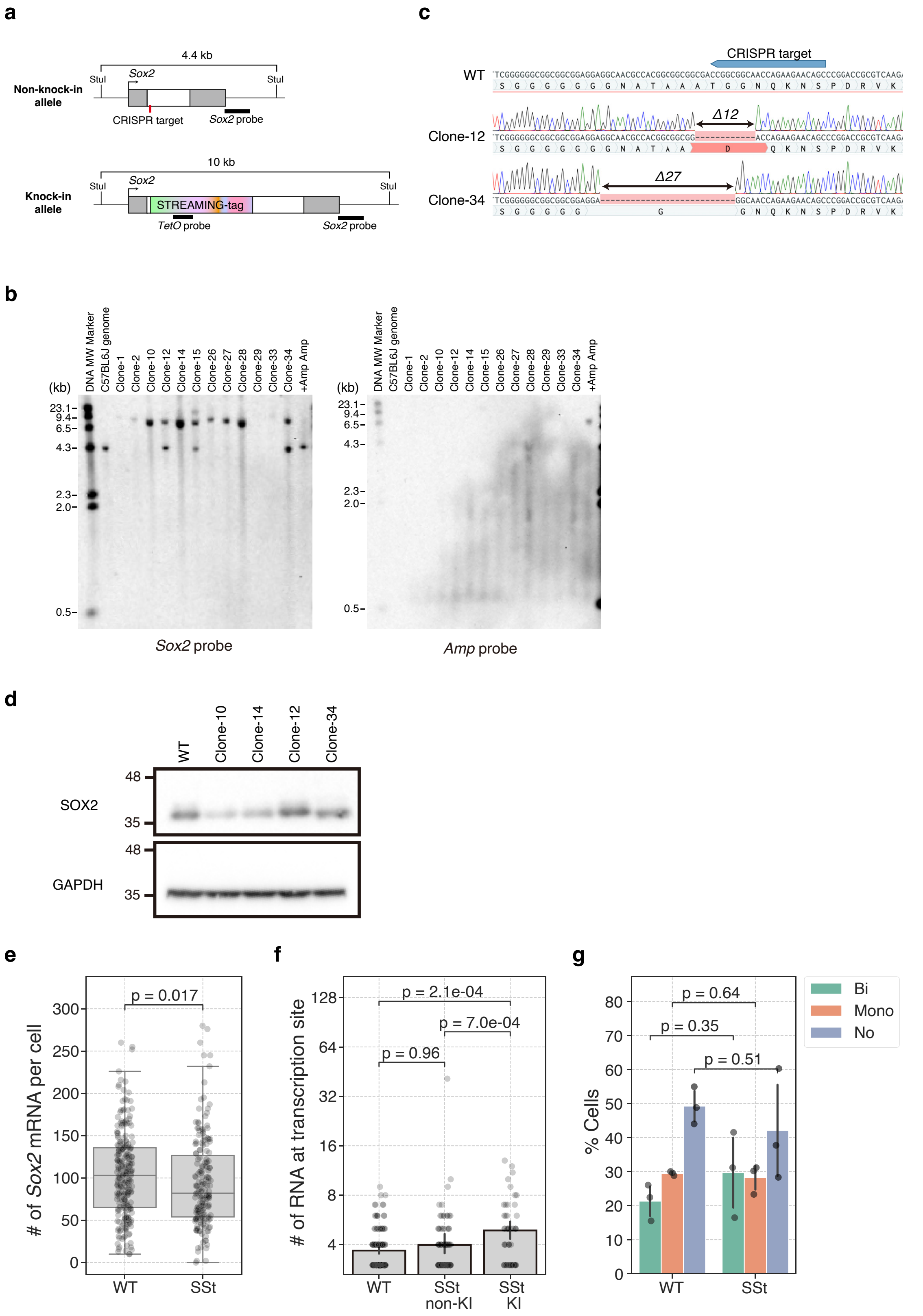

Supplementary Fig. 2 Establishment of *Sox2* STREAMING-tag knock-in cell lines. **a** Gene structure of mouse *Sox2* before and after STREAMING-tag knock-in. **b**

Southern blot analysis of *Sox2* STREAMING-tag knock-in candidate cells. **c** Sequence confirmation of non-knock-in allele in monoallelic knock-in candidate cells. In this study, we used Clone-12 as a *Sox2* STREAMING-tag knock-in cell. **d** Western blot analysis using SOX2 and GAPDH antibodies in knock-in candidate cells. Clone-10 and Clone-14 cells are biallelic, and Clone-12 and Clone-34 are monoallelic knock-in cells. **e** Distribution of *Sox2* mRNA counts in WT and *Sox2* STREAMING-tag knock-in (SSt) cells. WT, n = 246 cells; SSt, n = 187 cells. Box plots indicate the interquartile range IQR (25–75%) with a line at the median. Whiskers indicate 1.5 times the IQR. *P*-values were determined using two-sided Wilcoxon rank sum test. **f** Bar graph showing the mean number of RNA molecules at transcription sites in WT cells (WT), non-knock-in alleles of SSt cells (SSt non-KI), and knock-in alleles of SSt cells (SSt KI). Error bars indicate standard deviations. WT, n = 186, SSt non-KI, n = 131, SSt KI, n = 57. *P*-values were determined using two-sided Wilcoxon rank sum test. **g** Bar graph showing the frequency of cells with transcription sites in WT and SSt cells. Cells were classified as having no transcription sites (No), only one (Mono) or two (Bi). Data are presented as the means of n = 3 biological replicates (more than 50 cells per experiment), and error bars indicate standard deviations. *P*-values correspond to unpaired, two-sided Student's *t*-test.

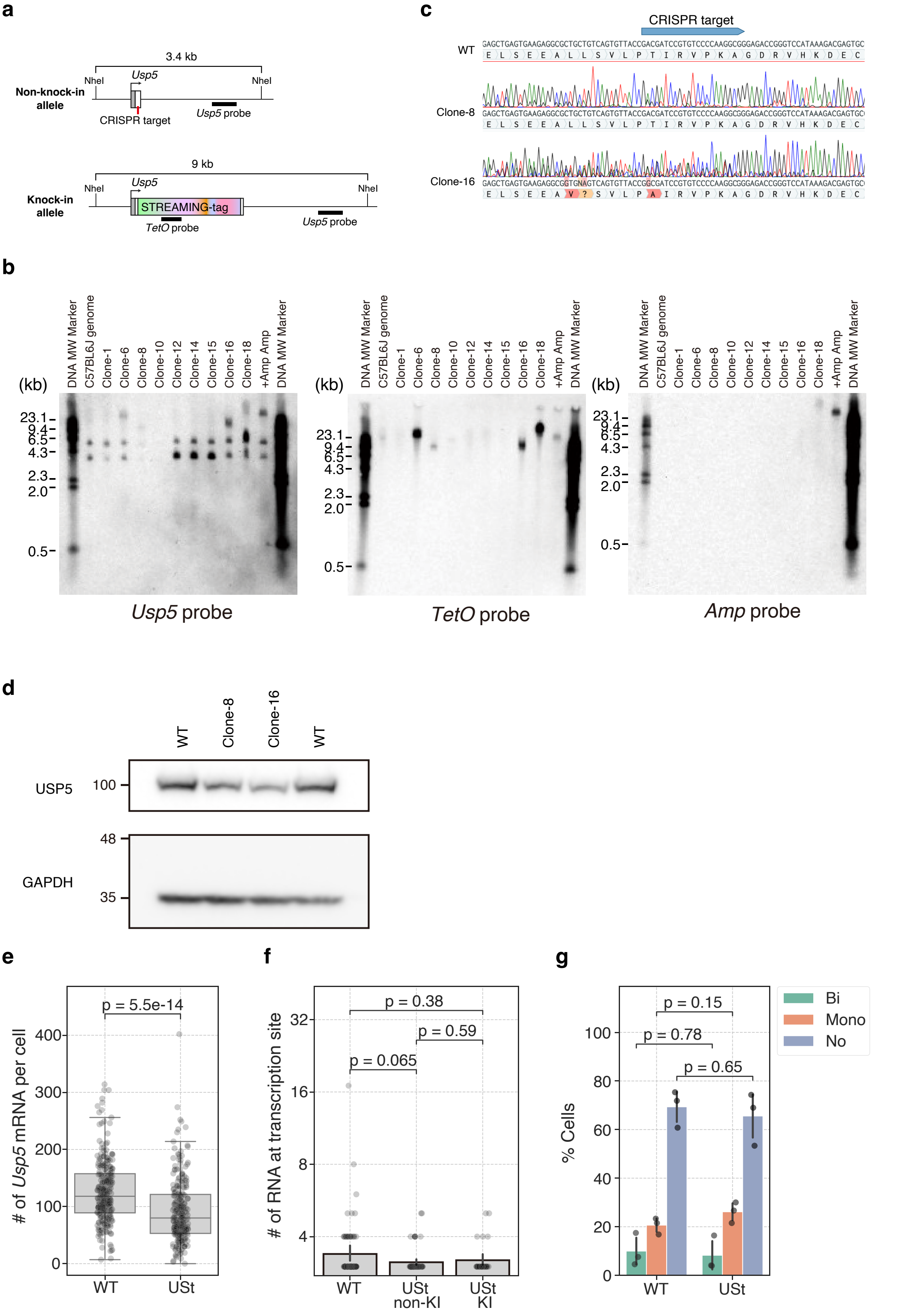

Supplementary Fig. 3 Establishment of *Usp5* STREAMING-tag knock-in cell lines.
a Gene structure of mouse *Usp5* before and after STREAMING-tag knock-in. **b**

Southern blot analysis of *Usp5* STREAMING-tag knock-in candidate cells. **c** Sequence confirmation of non-knock-in allele in monoallelic knock-in candidate cells. In this study, we used Clone-8 as a *Usp5* STREAMING-tag knock-in cell. **d** Western blot analysis using USP5 and GAPDH antibodies in knock-in candidate cells. **e** Distribution of *Usp5* mRNA counts in WT and *Usp5* STREAMING-tag knock-in (USt) cells. WT, *n* = 248 cells; USt, *n* = 267 cells. Box plots indicate the interquartile range IQR (25–75%) with a line at the median. Whiskers indicate 1.5 times the IQR. *P*-values were determined using two-sided Wilcoxon rank sum test. **f** Bar graph showing the mean number of RNA molecules at transcription sites in WT cells (WT), non-knock-in alleles of USt cells (USt non-KI), and knock-in alleles of USt cells (USt KI). Error bars indicate standard deviations. WT, *n* = 119, USt non-KI, *n* = 78, USt KI, *n* = 37. *P*-values were determined using two-sided Wilcoxon rank sum test. **g** Bar graph showing the frequency of cells with transcription sites in WT and USt cells. Cells were classified as having no transcription sites (No), only one (Mono) or two (Bi). Data are presented as the means of *n* = 3 biological replicates (more than 65 cells per experiment), and error bars indicate standard deviations. *P*-values correspond to unpaired, two-sided Student's *t*-test.

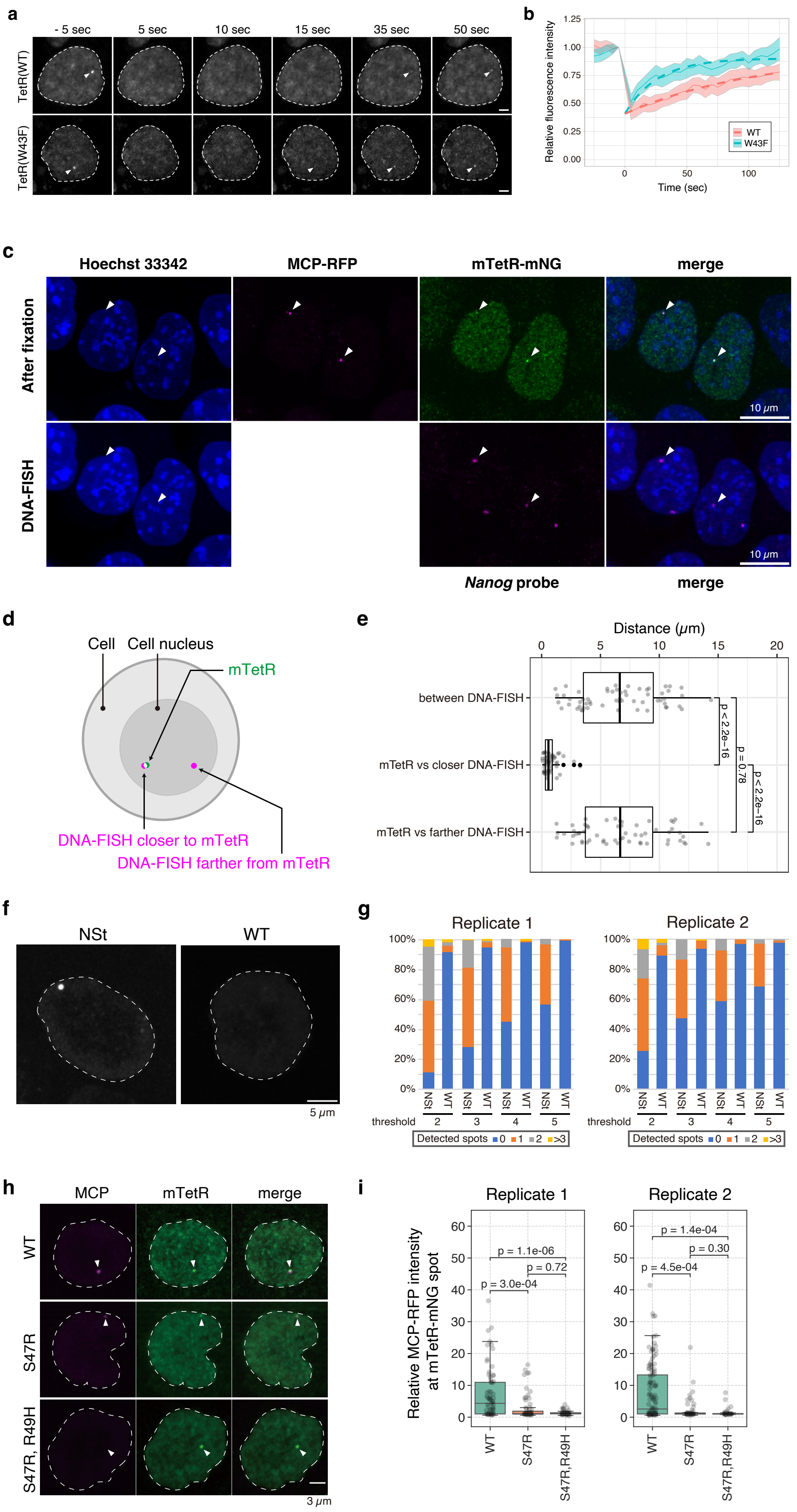
a
- 5 sec
5 sec
10 sec
15 sec
35 sec
50 sec
TetR(WT)
TetR(W43F)
b
Relative fluorescence intensity
Time (sec)
WT
W43F
c
Hoechst 33342
MCP-RFP
mTetR-mNG
merge
After fixation
DNA-FISH
10 μm
Nanog probe
merge
d
Cell
Cell nucleus
mTetR
DNA-FISH closer to mTetR
DNA-FISH farther from mTetR
e
Distance (μm)
between DNA-FISH
mTetR vs closer DNA-FISH
mTetR vs farther DNA-FISH
p < 2.2e−16
p = 0.78
p < 2.2e−16
f
NSt
WT
5 μm
g
Replicate 1
Replicate 2
threshold
Detected spots 0 1 2 >3
h
MCP
mTetR
merge
WT
S47R
S47R, R49H
3 μm
i
Replicate 1
Replicate 2
Relative MCP-RFP intensity at mTetR-mNG spot
p = 1.1e-06
p = 0.72
p = 3.0e-04
p = 1.4e-04
p = 0.30
p = 4.5e-04
WT
S47R
S47R,R49H

Supplementary Fig. 4. Specificity verification of target gene labeling in the STREAMING-tag system. a FRAP images. TetR-mNG spots were transiently bleached, and the recovery of spots was observed over time. Dashed lines and arrowheads indicate cell nuclei and TetR-mNG spots, respectively. Scale bar, 2 µm. **b** FRAP curves. Relative fluorescence intensities of TetR(WT)- and TetR(W43F)-mNG after photobleaching. Means ± 95% CI (WT, n = 24; W43F, n = 23) are shown as the fitted curves (bold dashed lines) with single exponential fitting with baseline (see Methods in detail). **c** DNA-FISH analysis. *Nanog* STREAMING-tag knock-in cell line (NSt)-derived cells expressing MCP-RFP and TetR(W43F)-mNG were imaged immediately after fixation with 4% paraformaldehyde. The same samples were subjected to DNA-fluorescence *in situ* hybridization using probes against the *Nanog* locus. Arrowheads indicate TetR(W43F)-mNG spots in close proximity to MCP-RFP and *Nanog* DNA-FISH spots. **d** Schematic diagram showing the positional relationship between the mTetR-mNG and DNA-FISH spots. In NSt cells, STREAMING-tag is monoallelically knocked in to *Nanog*. Therefore, two DNA-FISH spots are observed in the cell, but the mTetR spot is close to only one of them. **e** Distribution of distances between mTetR and DNA-FISH spots. The distance between two DNA-FISH spots (between DNA-FISH), the distance between a DNA-FISH spot closer to the mTetR spot and the mTetR spot (mTetR vs closer DNA-FISH), and the distance between another DNA-FISH spot farther from the mTetR spot and the mTetR spot (mTetR vs farther DNA-FISH). n = 59. Box plots indicate the interquartile range IQR (25–75%) with a line at the median. Whiskers indicate 1.5 times the IQR. P-values were determined using two-sided Wilcoxon rank sum test. **f** Images of NSt and WT ES cells expressing MCP-RFP. Dashed line indicates the cell nucleus. Scale bar, 5 µm. **g** Graphs showing the relative frequency (100% stacked columns) of the number of detected MCP spots per cell varied depending on the threshold levels. Candidate MCP-RFP spots were detected in each cell using the local maxima method. The relative RFP fluorescent intensity of each MCP-RFP spot relative to the cell nucleus was calculated. The number of MCP spots with relative fluorescent intensities of 2, 3, 4, 5, or higher are shown. Data derived from the two biological replicates are shown (more than 100 cells per experiment). **h** Representative images of cells derived from NSt cells and expressing mTetR-GFP and either MCP-RFP, MCP(S47R)-RFP or MCP(S47R, R49H)-RFP. Filled arrowheads indicate mTetR spot locations. Dashed line indicates the cell nucleus. Scale bar, 5 µm. **i** Distribution of RFP relative fluorescence intensity on mTetR-mNG spots, calculated as the relative intensity of RFP fluorescence on mTetR-mNG spots and in cell nuclei; results from two independent experiments are displayed. MCP, n = 72 (125);

MCP(S47R), n = 68 (58); MCP(S47R_R49H), n = 65 (40). Numbers in parentheses indicate the n of replicate 2. Box plots indicate the interquartile range IQR (25–75%) with a line at the median. Whiskers indicate 1.5 times the IQR. *P*-values were determined using two-sided Wilcoxon rank sum test.

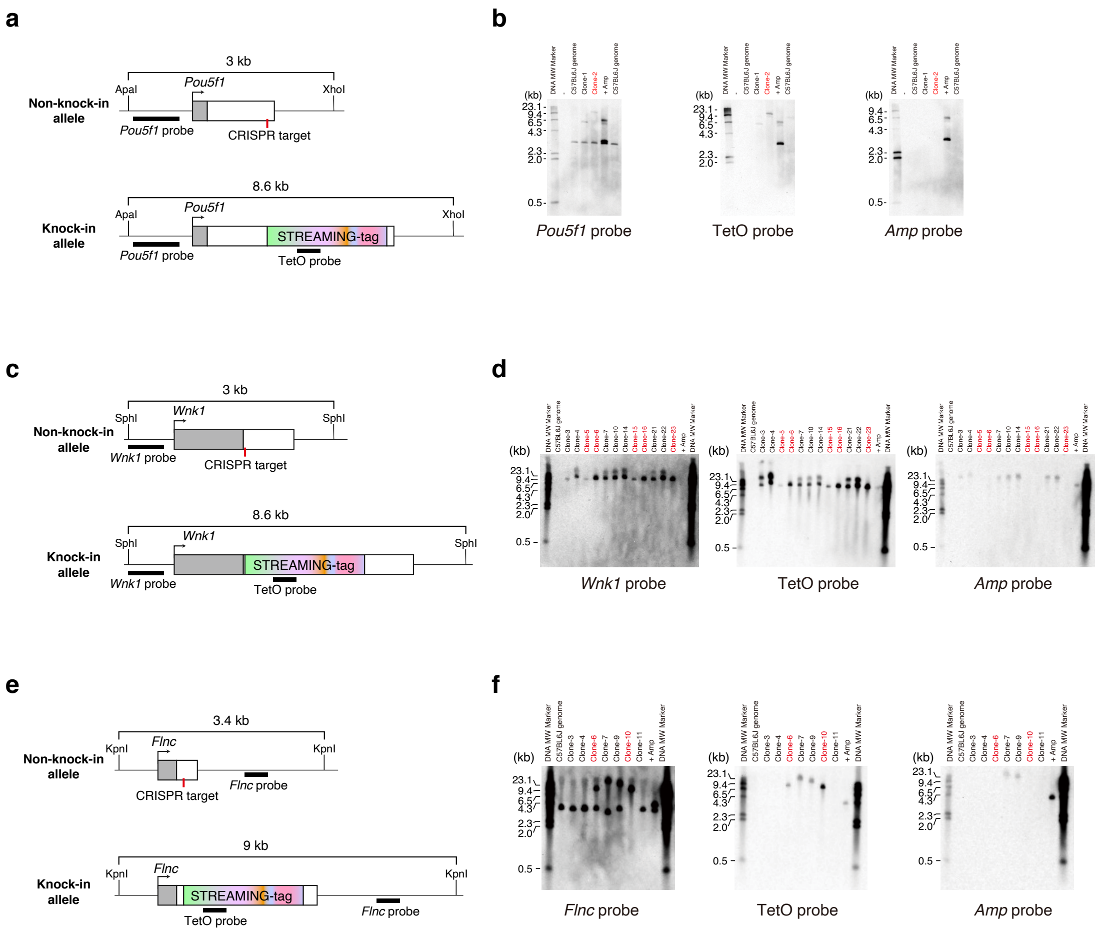

Supplementary Fig. 5 Establishment of *Pou5f1*, *Wnk1*, and *Flnc* STREAMING-tag knock-in cell lines. a Gene structure of mouse *Pou5f1* before and after STREAMING-tag knock-in. **b**, Southern blot analysis of *Pou5f1* STREAMING-tag knock-in candidate cells. Clones marked with red text indicate those that may not have random integration and have the desired cassette inserted in the expected position. We used Clone-2 as a *Pou5f1* STREAMING-tag knock-in cell. **c** Gene structure of mouse *Wnk1* before and after STREAMING-tag knock-in. **d** Southern blot analysis of *Wnk1* STREAMING-tag knock-in candidate cells. Clones marked with red text indicate clones that may not have random integration and have the desired cassette inserted in the expected position. In this study, we used Clone-6 as *Wnk1* STREAMING-tag knock-in cells. **e** Gene structure of mouse *Flnc* before and after STREAMING-tag knock-in. **f** Southern blot analysis of *Flnc* STREAMING-tag knock-in candidate cells. Clones marked with red text indicate those that may not have undergone random integration and have the desired cassette inserted in the expected position. We used Clone-6 as *Wnk1* STREAMING-tag knock-in cells.

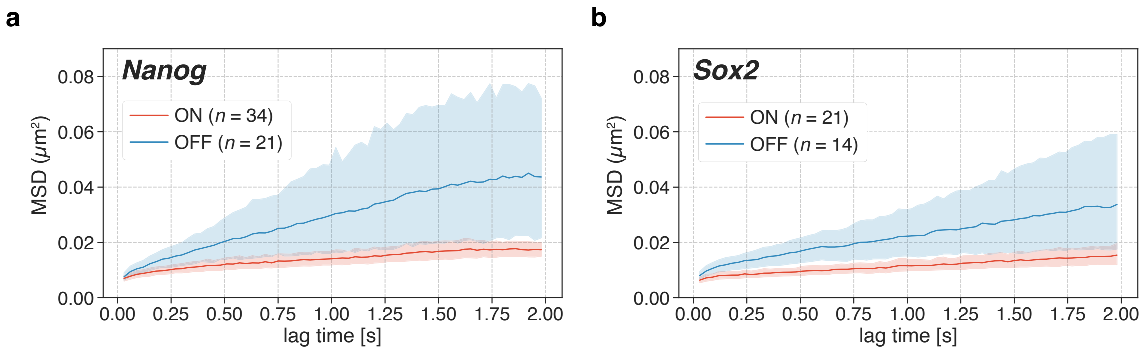

Supplementary Fig. 6. MSD plots of *Nanog* and *Sox2* in mESCs. a-b MSD of mTetR spots in NSt-NLS-SNAP cell line derived from *Nanog* STREAMING-tag knock-in cell line, which stably expresses NLS-SNAPtag, for *Nanog* (**a**) and the SSt-NLS-SNAP cell line derived from *Sox2* STREAMING-tag knock-in cell line, which stably expresses NLS-SNAPtag, for *Sox2* (**b**). The data are classified into the ON and OFF states. Means ± 95% CI are shown. *n*, number of cells analyzed. The plots were fitted as a subdiffusive curve: MSD = $4D_\alpha t^\alpha$, where D_α is the apparent diffusion coefficient with the physical dimension $(\mu m)^2/(t)^\alpha$, and α is the anomalous coefficient. The D_α of *Nanog* in the ON and OFF states and of *Sox2* in the ON and OFF states were 0.0037, 0.0077, 0.0030, and 0.0059, respectively. The α of *Nanog* in the ON and OFF states and of *Sox2* in the ON and OFF states were 0.25, 0.49, 0.23, and 0.37, respectively.

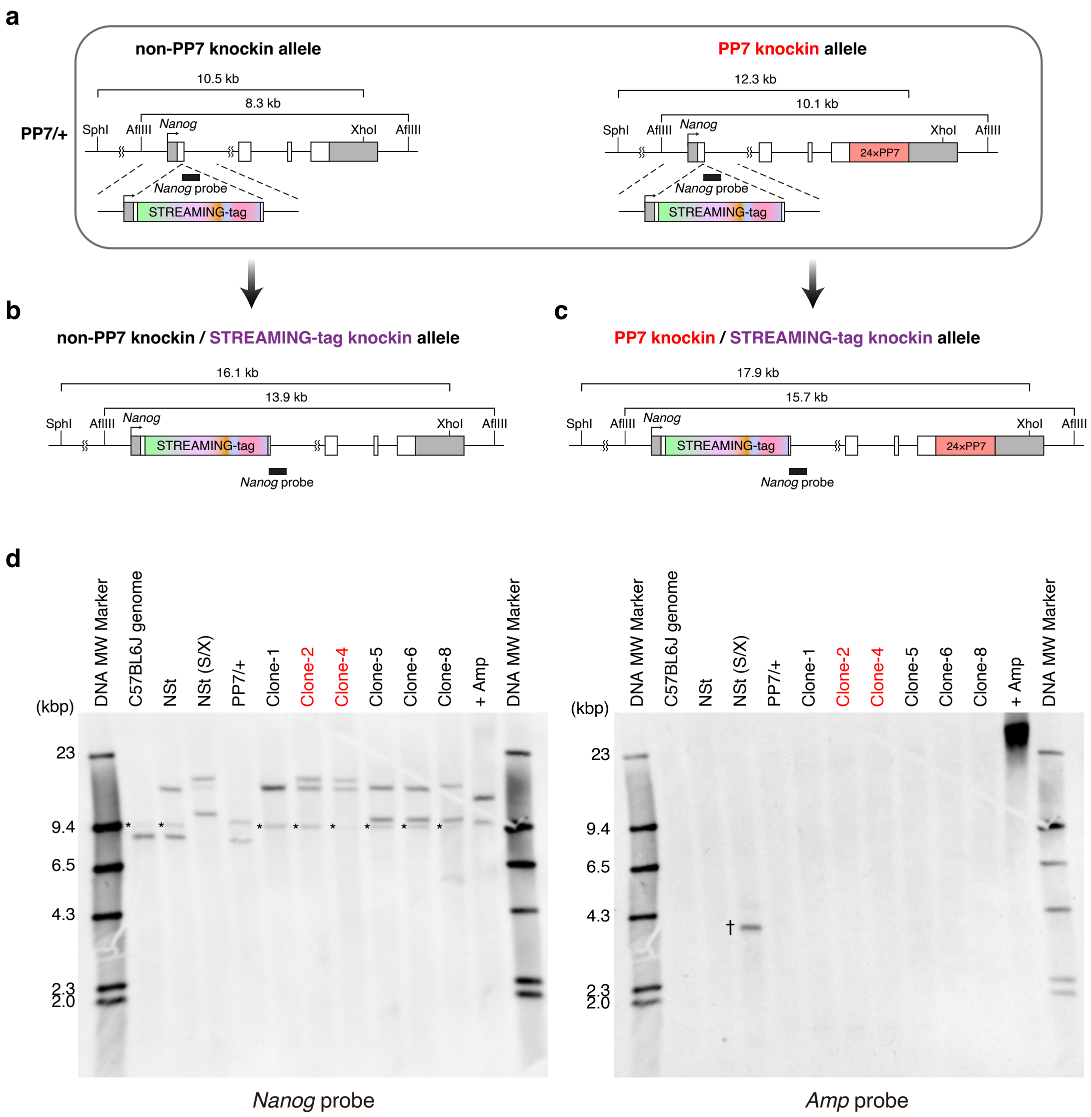

Supplementary Fig. 7. Establishment of STREAMING-tag/PP7 knockin cell lines. **a** Gene structure of *Nanog* in PP7/+ cell line in which a PP7 repeat was monoallelically knocked into the *Nanog* 3' UTR. **b** Gene structure of *Nanog* STREAMING-tag knockin allele. **c** Gene structure of *Nanog* STREAMING-tag and PP7 knockin allele. **d** Southern blot analysis of *Nanog* STREAMING-tag and PP7 knock-in candidate cells. For Southern blot analysis, SphI/XhoI were used in samples marked S/X, while AflIII were used for others. NSt, *Nanog* STREAMING-tag knockin cell line. Clones marked with red text indicate those that may not have random integration and have the *Nanog* allele in which STREAMING-tag and PP7 repeat were knocked in the expected position. Asterisks indicate nonspecific bands. The dagger symbol indicates unintended band of unknown origin; the genomic DNA of NSt cells is unlikely to contain the *Amp* gene because they are not detected when treated with AflIII. In Clone-2 and -4, a PP7 repeat

was monoallelically knocked into the *Nanog* 3' UTR and STREAMING-tags were biallelically knocked into TSS-proximal region of *Nanog*. We used Clone-4 as a *Nanog* STREAMING-tag and PP7 knock-in cell line (*Nanog*-STREAMING-PP7).

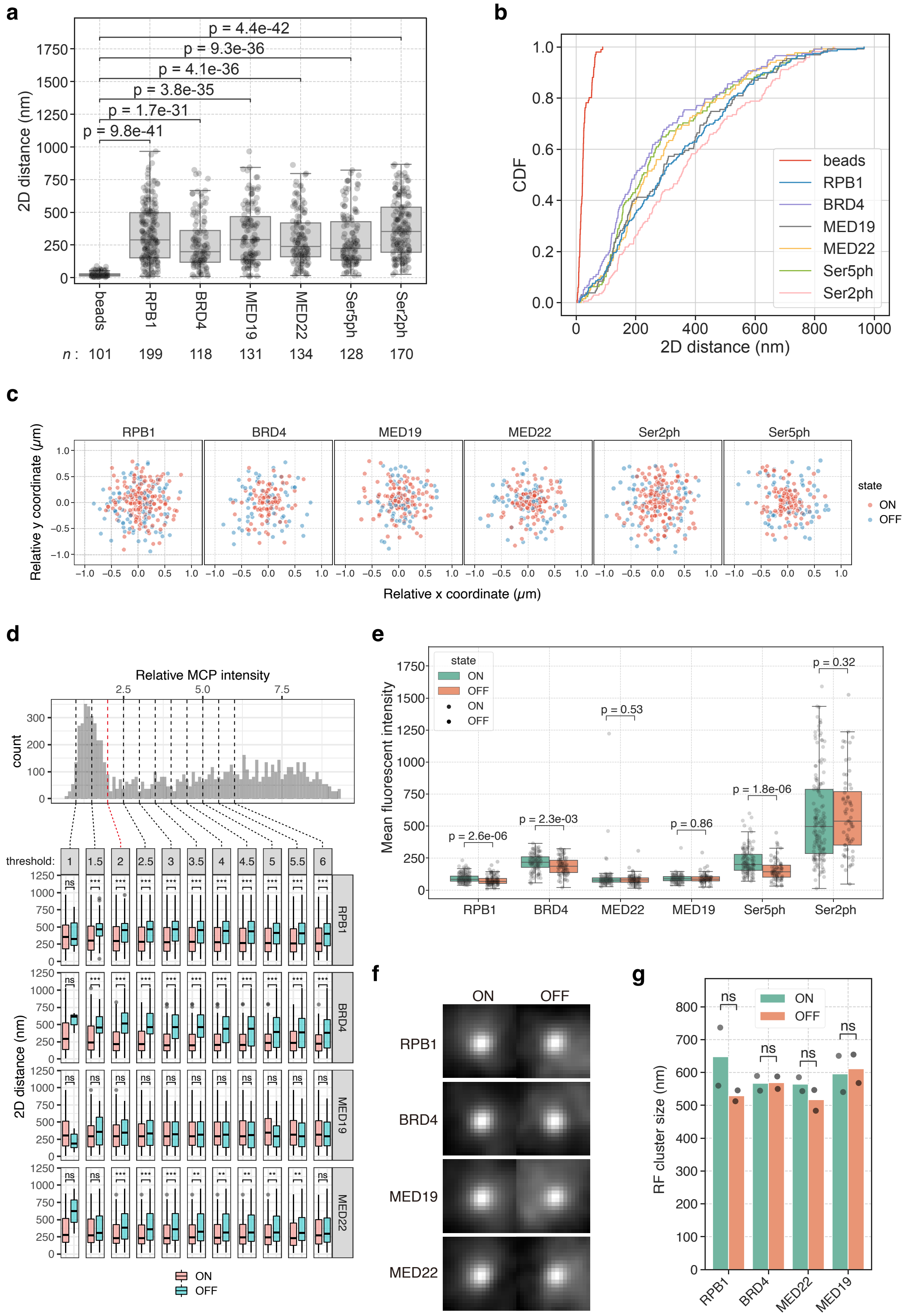

Supplementary Fig. 8. Distribution of regulatory factor (RF) cluster-mTetR distance in *Nanog* STREAMING-tag knock-in cells. a, 2D distances between mTetR

spots and the nearest RF clusters in the ON states. *n*, number of cells analyzed. To investigate chromatic aberrations between the mNG and SNAPtag channels of the microscope used, the spots of the mNG/SNAPtag channels of 0.1 μm fluorescent beads (27 ± 19 nm in 2D) were measured. Box plots indicate the interquartile range IQR (25–75%) with a line at the median. Whiskers indicate 1.5 times the IQR. The distances between all mTetR-RF clusters were significantly larger than the measurement error. *P*-values were determined using two-sided Wilcoxon rank sum test. **b**, Cumulative density function (CDF) of the data in (**a**) is shown. **c**, Relative mTetR-RF-cluster xy coordinates. Data are classified by transcriptional states. RPB1, *n* = 199 (115); BRD4, *n* = 118 (73); MED19, *n* = 131 (75); MED22, *n* = 134 (83); Ser5ph, *n* = 128 (73); Ser2ph, *n*= 170 (82), at the ON state (OFF state). **d** Robustness of threshold settings for ON/OFF state classifications. Box plots indicate the interquartile range IQR (25–75%) with a line at the median. Whiskers indicate 1.5 times the IQR. The upper panel shows the distribution of relative MCP intensity in cells with SNAPtag knock-in for RPB1, BRD4, MED19, or MED22. The lower panel shows the distribution of the distance between mTetR and the nearest RF clusters for each state, judged as ON if it is higher than the presented threshold and OFF if it is lower. Statistical significance was assessed by Wilcoxon rank sum and indicated by asterisks. A single asterisk (*) represents $p \leq 0.05$, double asterisk (**) represents $p \leq 0.01$, and triple asterisk (***) represents $p \leq 0.001$, with ns indicating no significant difference. RPB1, *n* = 314; BRD4, *n* = 191; MED19, *n* = 206; MED22, *n* = 217. The result remained essentially the same by varying the threshold from 2 to 5.5, indicating the robustness of the analysis. We kept the 2-fold threshold data in the main Figs. **e** Distribution of mean fluorescence intensities for RF clusters in the nearest neighbor of *Nanog*. Box plots indicate the interquartile range IQR (25–75%) with a line at the median. Whiskers indicate 1.5 times the IQR. The values of *N* are the same as in panel **c**. *P*-values were determined using two-sided Wilcoxon rank sum test. **f** Averaged images of RF clusters in the nearest neighbor of *Nanog* in the ON or OFF state. **g** Bar graph of the size of RF clusters in the nearest neighbor of *Nanog* in the ON or OFF state. The full width at half maximum is calculated based on the image in Panel e (see Methods for details). Data are presented as the means of *n* = 2 biological replicates (more than 30 clusters per experiment). *P*-values correspond to unpaired, two-sided Student's *t*-test.

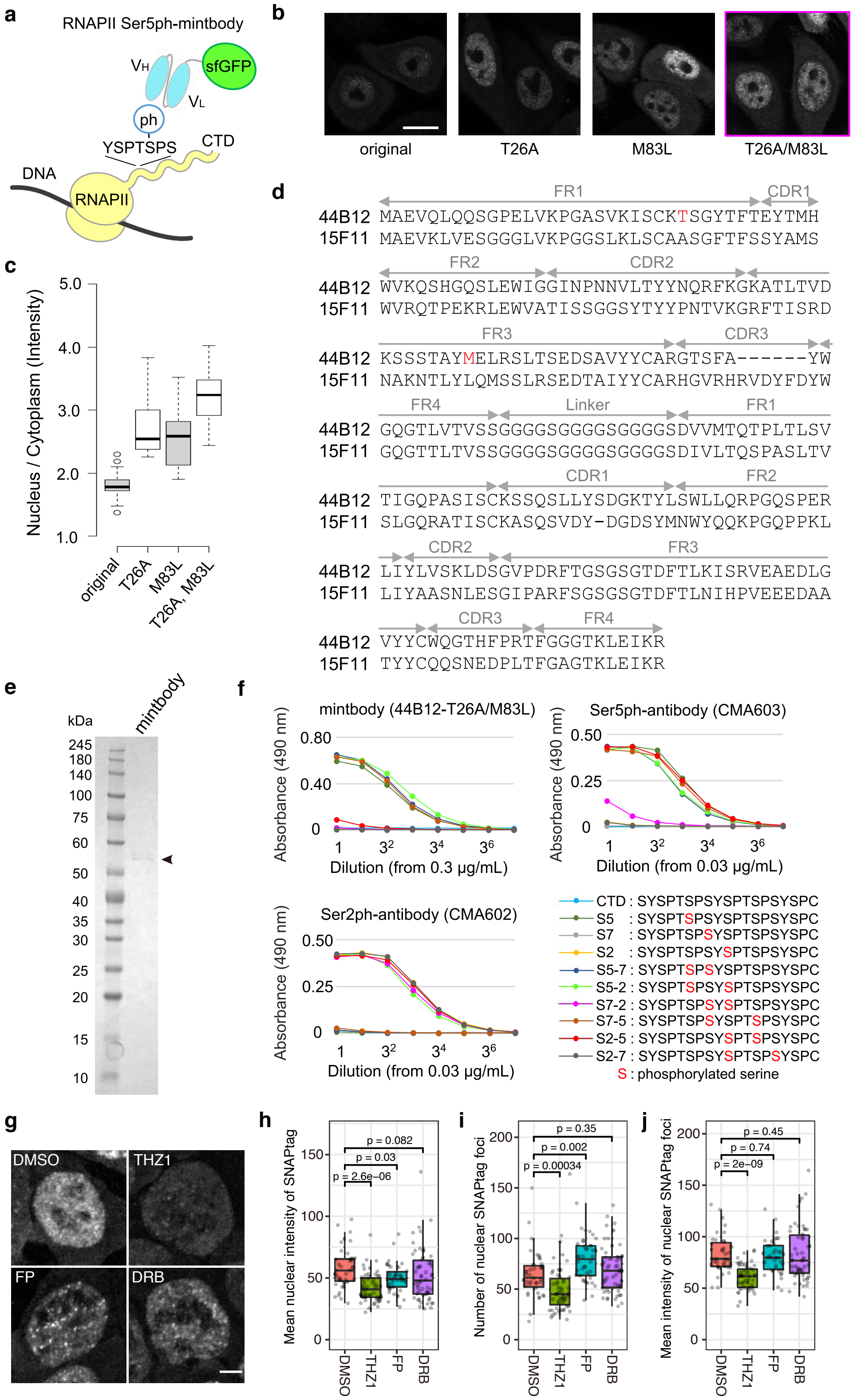

Supplementary Fig. 9 Establishment of a mintbody that recognizes RNAPII Ser5ph. **a**, Schematic representation of the mintbody that binds to phosphorylated Ser5 on the C-terminal domain of RNA polymerase II (RNAPII). **b**, Example images of

mutant scFv-sfGFP stably expressed in HeLa cells. The image acquisition and contrast adjustment settings were the same, enabling comparison of mintbody localization between samples. Scale bar, 10 µm. **c**, Nuclear-to-cytoplasm fluorescence intensity ratios of 44B12 and mutants. HeLa cells expressing 44B12-sfGFP and mutants were established. After confocal image acquisition, the nuclear-to-cytoplasmic fluorescence intensity ratios were measured (*n* = 20 cells). Box plots indicate the interquartile range IQR (25–75%) with a line at the median. Whiskers indicate 1.5 times the IQR. **d**, Comparison of amino acid sequences of RNAPII Ser5ph (44B12) and H4K20me1 (15F11) scFv. The framework region (FR), complementarity determining region (CDR), and linker region are shown. Mutation sites in 44B12 are indicated in red letters. The final construct, named as RNAPII Ser5ph-mintbody, contains T26A and M83L substitutions. **e** and **f**, His-tagged RNAPII Ser5ph-mintbody was expressed in *Escherichia coli*, purified using an Ni-column, treated with enterokinase to remove the His-tag, and further purified. **e**, Purified proteins were separated on an SDS-polyacrylamide gel and stained with Coomassie blue. The positions of the size marker are shown on the left. **f**, Enzyme-linked immunosorbent assay plates coated with synthetic peptides conjugated with bovine serum albumin were incubated with a dilution series of purified RNAPII Ser5ph-mintbody and control antibodies specific for RNAPII Ser2ph (CMA602) and RNAPII Ser5ph (CMA603). After incubation with peroxidase-conjugated anti-GFP (for RNAPII Ser5ph-mintbody) or anti-mouse IgG (for monoclonal antibody) and then with o-phenylenediamine, the absorbance was measured at 490 nm. RNAPII Ser5ph-mintbody reacted with peptides containing Ser5ph. **g**, Images showing Nst-SNAP-Ser5ph cells stably expressing RNAPII Ser5ph mintbody-SNAPtag cultured with transcriptional inhibitors. Nst-SNAP-Ser5ph cells were labeled with SNAP-Cell 647-SiR and cultured for 1 h either with 15 µM THZ1, 1 µM flavopiridol (FP), 100 µM 5,6-dichloro-1-β-d-ribofuranosylbenzimidazole (DRB) or dimethyl sulfoxide (DMSO) as a vehicle. Scale bar, 5 µm. **h-j**, Boxplots with all data points showing mean intensity of RNAPII Ser5ph-mintbody in the nucleus **h**, number of RNAPII Ser5ph-mintbody foci in the nucleus (**i**), and mean intensity of RNAPII Ser5ph-mintbody foci in each cell (**j**). Box plots indicate the interquartile range IQR (25–75%) with a line at the median. Whiskers indicate 1.5 times the IQR. *P*-values were determined using two-sided Wilcoxon rank sum test. DMSO, *n* = 39; THZ1, *n* = 55; FP, *n* = 38; DRB, *n* = 54.

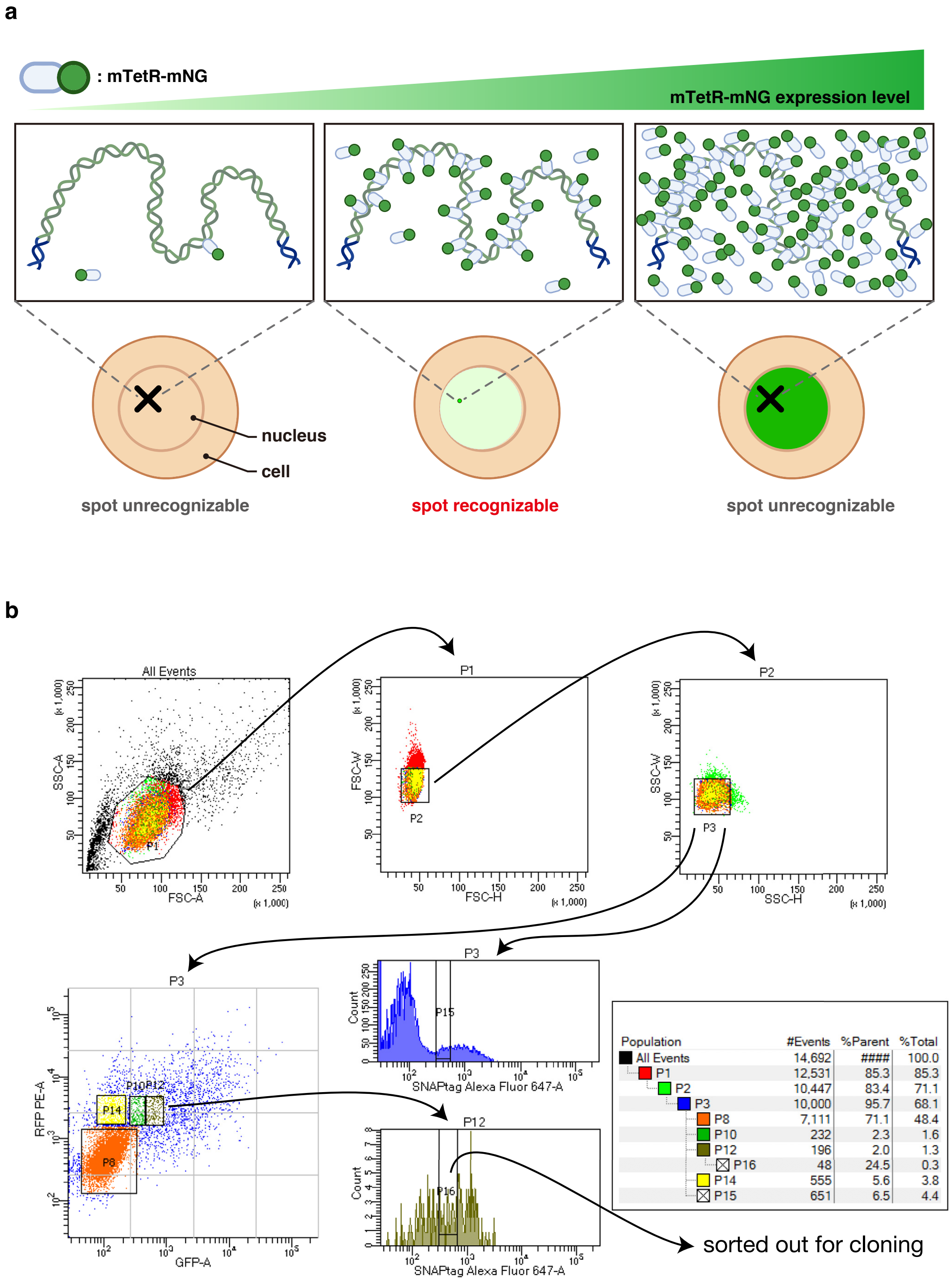

Supplementary Fig. 10. Method for establishing cells that moderately express mTetR-mNG, MCP-RFP, and mintbody-SNAP-tag. mTetR-mNG binds specifically to the TetO sequence. By inserting TetO repeats into a specific genomic region and expressing mTetR-mNG, the region can be visualized. This procedure can also be used for MS2 repeat/MCP-RFP and RNAPII Ser2ph or Ser5ph/mintbody. However, the expression levels of fluorescent protein fusion proteins should be carefully monitored.

a, Visualization of the TetO repeat requires an appropriate amount of mTetR-mNG expression. If the amount of mTetR-mNG is critically low, the number of mTetR-mNG molecules that bind to the target region will be low, making it difficult to recognize the mTetR spot using fluorescence microscopy (left panel). If there is excess mTetR-mNG, the number of mTetR-mNG molecules bound to the target region will be high. However, if the concentration of unbound mTetR-mNG in the cell nucleus is higher than the local concentration of mTetR-mNG bound to TetO repeats, recognition of the mTetR spot is difficult (right panel). If the appropriate amount of mTetR-mNG is expressed, the local concentration of mTetR-mNG bound to TetO repeats will be higher than that of unbound mTetR-mNG in the cell nucleus, allowing for recognition of the mTetR spot (middle panel). Therefore, it is necessary to select cells expressing an appropriate amount of mTetR-mNG. The same procedure can be used for MS2 repeat/MCP-RFP and RNAPII Ser2ph or Ser5ph mintbody. **b**, Fluorescence-activated cell sorting (FACS) gating strategy for cells expressing appropriate amounts of mTetR-mNG, MCP-RFP, and mintbody-SNAPtag in mouse embryonic stem cells (mESCs). First, we prepared cells stably expressing mTetR-mNG, MCP-RFP, and mintbody-SNAPtag using the piggyBac system in mESCs (see Methods). From these cell populations, cells expressing appropriate amounts of mTetR-mNG, MCP-RFP, and mintbody-SNAPtag were sorted using FACS. Shown is an example of an FACS gating strategy in mTetR-mNG-, MCP-RFP-, and RNAPII Ser2ph mintbody-SNAPtag-expressing cells. Immediately before FACS, the cells were treated with SNAP-Cell 647-SiR and stained with SNAPtag. The P8 gate is a cell population that does not express mNG or RFP. Approximately, 300 cells from the P16 gate were sorted using FACS and plated into 6 cm dishes. The resulting colonies were passaged on an 8-well chambered cover glass with #1.5 glass (Cellvis, catalog number: C8-1.5HN) for microscopic observation, and cells expressing the appropriate amount of mNG/RFP/SNAPtag were selected using microscopy.

Supplementary Table 1. Primer sequence for RNAPII ChIP-qPCR.

Region name	Forwad	Reverse
R1	TCCTGGTCCCCACAGTTTGCCT	CGAATTCGCGGCCGCTAACCAA
R2	TGACGCTTGGCTGCTGACAACC	ACTGTGGAGCCGCCTGAGGAAT
R3	AGGTGGAAGTGGTGGGGGAGTA	TGCTTGCAACCAGACCATTCTTCAGG
Gapdh promoter	TGCCCACTCCCCTTCCCAGTTT	TCCAGAGACGGCCGCATCTTCT

Supplementary Methods 1: Detailed step-by-step protocols

Construction of STREAMING-tag knock-in vectors

Materials:

- Left-arm-F primer (see below)
- Left-arm-R primer (see below)
- Right-arm-F primer (see below)
- Right-arm-R primer (see below)
- KOD One polymerase (TOYOBO, KMM-101X5) or another high-fidelity DNA polymerase
- Agarose S (Nippon Gene, 313-90231)
- TBE buffer (Takara Bio, T9122)
- Zymoclean™ Gel DNA Recovery Kits (Zymo Research, D4002)
- BsaI-HFv2 (NEB, R3733S)
- SacI-HF (NEB, R3156S)
- KpnI-HF (NEB, R3142S)
- pBSKΔBS-SD_MCS_SA(NBSI)-24xMS2-sirius-96xopto-TetO-Int-3xFLAG-NeoR-GS4 (Addgene ID # 177265).
- pBSKΔB (Addgene ID # 177268)
- NEBuilder HiFi DNA Assembly kit (NEB, E2621L)
- One Shot™ Stbl3™ Chemically Competent (Thermo Fisher Scientific, C737303)
- LB medium (Nacalai Tesque, 20068-75)
- Ampicillin (Nacalai Tesque, 02739-74)
- GenElute HP Plasmid Miniprep Kit (Sigma-Aldrich, NA0160-1KT)

Protocols:

1. Determine the knock-in site of the STREAMING-tag cassette based on the genome sequence of the target species. Ideally, the knock-in site should be near the transcription start site (TSS) and translated by all transcripts. Considering the following points, it is necessary to knock-in at appropriate sites in the coding region near the TSS. First, it has been reported that the length of the first exon may affect transcriptional activity[1]. As the splice donor is just below the 5′ side of the STREAMING-tag cassette, the exon length may be altered by the STREAMING-tag knock-in. Moreover, because of the N-end rule, knock-in of the STREAMING-

tag immediately after the initiation codon should be avoided[2]. Thus, it is considered to knock-in the STREAMING-tag at least three amino acids downstream from the starting methionine. In addition, some proteins may contain a domain near the N-terminus, which is important for protein function. As STREAMING-tag knock-in results in insertion of an amino acid sequence, QGCF (Supplementary Fig. 1c), the knock-in position should be determined on a case-by-case basis. Because the 5′ and 3′ ends of the STREAMING-tag both encode amino acids (Supplementary Fig. 1c), the knock-in site should be set between the codon sequences to be in-frame. In addition, there must be a highly specific CRISPR target sequence that overlaps the desired knock-in position. For mice and humans, highly specific CRISPR target sequences can be easily identified using the UCSC genome browser (http://genome.ucsc.edu). In addition, the insertion site should be designed to be located 10–20 bases from the spacer sequence of the CRISPR target sequence, which will prevent the CRISPR from being re-cut after knock-in.
Once the insertion position was determined, the primer sets were designed. The following primer set was used to target the *Sox2* locus:
Left-arm-F (5′-CTATAGGGCGAATTGGGTACGAGCGCAGTGCCGCGGATGAGCGC-3′), Left-arm-R (5′-GATCGAATACTTATCGCTTACCTGGCCGGTCGCCGCCGCCGTGGCGTT-3′), Right-arm-F (5′-GGTCTGGTTGCAAGCAATTGCTTCGGCAACCAGAAGAACAGCCCGGAC-3′), and Right-arm-R (5′-AGGGAACAAAAGCTGGAGCTAATGGGCCTTAAAAATACCAGCGG-3′).

2. Red letters indicate homologous sequence sites between fragments that will be used when assembling with NEBuilder, and underlined green letters indicate additional sequences for codon frame adjustment. Depending on the target sequence, the black part can be modified appropriately. We designed the left and right arms to be approximately 1 kbp in length. NCBI Primer-BLAST (https://www.ncbi.nlm.nih.gov/tools/primer-blast/) was used for primer design. These oligo DNAs can be synthesized by an appropriate company.
3. Amplify the left arm and right arm separately by PCR using high-fidelity thermostable DNA polymerase (such as KOD One polymerase, TOYOBO, Osaka, Japan) using the prepared oligo DNA and genomic DNA of the target species as templates.
4. Resolve the PCR products on 1% agarose gel in 0.5× TBE buffer.

5. Cut out approximately 1 kbp fragments of the left and right arm fragments from the agarose gels.
6. Purify the DNA from the gel slabs using Zymoclean™ Gel DNA Recovery Kits and elute the DNA with 10 µL of ddH_2O. Measure the DNA concentration using a Nanodrop to ensure correct stoichiometry in the following steps. If not used, immediately freeze and store the DNA frozen until needed.
7. Treat the pBSKΔBS-SD_MCS_SA(NBSI)-24xMS2-sirius-96xopto-TetO-Int-3xFLAG-NeoR-GS4 (Supplementary Table 2) with BsaI. Resolve the digestion product on a 1% agarose gel in 0.5×TBE buffer.
8. Cut out the ~5.5 kbp fragment of pBSKΔBS-SD_MCS_SA(NBSI)-24xMS2-sirius-96xopto-TetO-Int-3xFLAG-NeoR-GS4 BsaI digest from the agarose gels.
9. Purify the DNA from the gel slabs using Zymoclean™ Gel DNA Recovery Kits and elute the DNA with 10 µL of ddH_2O. This fragment is named as the STREAMING-tag fragment. Measure the DNA concentration with a Nanodrop to ensure correct stoichiometry in the following steps. If not used, immediately freeze and store the DNA until needed.
10. Treat pBSKΔB (Supplementary Table 2) with SacI and KpnI. Resolve the digest on a 1% agarose gel in 0.5×TBE buffer.
11. Cut out the ~3 kbp fragment of pBSKΔB SacI/KpnI digest from the agarose gels.
12. Purify the DNA from the gel slabs using Zymoclean™ Gel DNA Recovery Kits and elute the DNA with 10 µL of ddH_2O. This fragment is named as the backbone fragment. Measure the DNA concentration using a Nanodrop to ensure the correct stoichiometry in the following steps. If not used, immediately freeze and store the sample until required.
13. Use the NEBuilder HiFi DNA Assembly kit to assemble these fragments together.
14. Prepare the fragments in advance by diluting them with ddH_2O to the following concentrations.

Fragments	Size (kbp)	Conc. (ng/µL)
Left arm fragment	1	3.335
Right arm fragment	1	3.335
Backbone fragment	3	5
STREAMING-tag fragment	5.5	18.3

15. Mix the samples in the following combinations: Prepare a reaction solution with only the backbone added as a negative control.

Reaction	Backbone	Inserts	Volume of backbone (μL)	Volume of insert (μL)	NEBuilder HiFi DNA Assembly Master Mix
Targeting vector	Backbone fragment	Left arm fragment<br>Right arm fragment<br>STREAMING-tag cassette	0.5	0.5<br>0.5<br>0.5	2
Control	Backbone fragment	H_2O	0.5	1.5	2

16. Incubate the sample in a thermocycler at 50°C for 60 min, and then place the samples on ice.
17. Transform the targeting vector and control reactions into 50 μL of Stbl3 chemically competent cells as per the manufacturer's protocol.
18. Spread *Escherichia coli* onto LB agarose medium containing ampicillin and incubate at 30°C overnight.
19. If the number of colonies does not exceed twice that of the control, most plasmids were not successfully created. In this case, it is recommended that the samples be reconditioned.
20. Seed the resulting colonies individually into 3 mL of LB medium containing ampicillin and incubate the cultures overnight at 32°C with shaking.
21. Collect the plasmid with the GenElute HP Plasmid Miniprep Kit.
22. Treat the recovered plasmid with appropriate restriction enzymes and verify the expected band pattern.
23. As the homology arm region amplified using PCR and junction sites in NEBuilder HiFi-assembled plasmids are prone to mutation, these regions should be confirmed using Sanger sequencing using appropriate oligo DNA.
24. The final plasmid should be purified using an appropriate method to achieve transfection-grade purity.

Construction of the CRISPR vector

Materials:

- eSpCas9-EF (Addgene ID # 177267)
- BbsI-HF (NEB, R3539S)
- Agarose S (Nippon gene, 313-90231)
- TBE buffer (Takara Bio, T9122)
- Zymoclean™ Gel DNA Recovery Kits (Zymo Research, D4002)
- Sense oligo (see below)
- Antisense oligo (see below)
- T4 Polynucleotide Kinase (PNK) (NEB, M0201S)

- ATP (NEB, P0756S)
- Ligation-Convenience Kit (Nippon gene, 319-05961)
- One Shot™ Stbl3™ Chemically Competent (Thermo Fisher Scientific, C737303)
- LB medium (Nacalai Tesque, 20068-75)
- Ampicillin (Nacalai Tesque, 02739-74)
- GenElute HP Plasmid Miniprep Kit (Sigma-Aldrich, NA0160-1KT)
- LKO1-5 primer (5′-GACTATCATATGCTTACCGT-3′)

Protocols:

1. Treat the eSpCas9-EF plasmid (Supplementary Table 2) with BbsI, and resolve the digest on a 1% agarose gel in 0.5× TBE buffer.
2. Cut out the ~8.5 kbp fragment of eSpCas9-EF BbsI digest from the agarose gels.
3. Purify the DNA from the gel slabs using Zymoclean™ Gel DNA Recovery Kits, and elute the DNA with 10 µL of ddH_2O. This fragment is named as the backbone fragment. Measure the DNA concentration with a Nanodrop to ensure the correct stoichiometry in the following steps. If not used, immediately store the fragment frozen until needed.
4. Order the following oligo DNA containing the spacer sequence corresponding to the CRISPR target sequence determined above (5′-GCTGTTCTTCTGGTTGCCGCCGG -3′ for Sox2; underlined part indicates the PAM sequence). Sense oligo: 5′-CACCGGCTGTTCTTCTGGTTGCCGC-3′, antisense oligo: 5′-AAACGCGGCAACCAGAAGAACAGCC-3′. The red letters are additional sequences for cloning, and underlined parts indicate an additional G base at the 5′ end of the spacer sequence. This additional G base is inserted because it is required for transcription initiation by the U6 promoter. If the 5′ end of the spacer sequence is G, this additional G base does not need to be inserted.
5. To perform phosphorylation and annealing of oligos, prepare the following reaction solutions in PCR tubes. Finally, add the relevant oligonucleotides.

Reagent	Volume (µL)
oligo 1 (50 µM)	0.5
oligo 2 (50 µM)	0.5
10X PNK Buffer (NEB)	0.5
T4 PNK (NEB)	0.2
10 mM ATP	0.5
ddH_2O	2.8

6. Mix the reaction solution well by tapping or pipetting, and collect the sample at the bottom of the tube by centrifugation.
7. Perform the following reaction in a thermal cycler to anneal and phosphorylate the oligos: 37°C for 30 min, 95°C for 5 min, and then ramp down to 25°C at 5°C/min.
8. After the reaction, collect the sample at the bottom of the tube by centrifugation and place the sample on ice. This fragment is named as the spacer fragment.
9. Remove the Ligation-Convenience Kit from the freezer, thaw if frozen, and place on ice.
10. Prepare the samples as follows: backbone should be pre-diluted as appropriate.

	**Backbone**	**Insert**	**Volume of backbone (μL)**	**Volume of insert (μL)**	**2x Ligation-Convenience Kit**
Test	Backbone fragment (2 ng/μL)	Spacer fragment (1/1800 diluted)	0.5	0.5	1
Control	Backbone fragment (2 ng/μL)	ddH_2O	0.5	0.5	1

11. Mix the reaction contents well by pipetting and allow to ligate at 16°C for 30 min.
12. Transform the sample into Stbl3 chemical competent cells according to the manufacturer's protocol.
13. Spread *Escherichia coli* onto LB agarose medium containing ampicillin, and incubate the cells at 37°C overnight.
14. If the number of colonies does not exceed twice that of the control, it is likely that most plasmids were not successfully prepared. In this case, it is recommended that the samples be reconditioned.
15. Place the resulting colonies individually into 3 mL of LB medium containing ampicillin, and incubate the cultures overnight at 37°C with shaking.
16. Purify the plasmid using the GenElute HP Plasmid Miniprep Kit.
17. Treat the recovered plasmid with appropriate restriction enzymes, and confirm that the expected band pattern is obtained.
18. As the homology arm region amplified using PCR and junction sites in NEBuilder HiFi-assembled plasmid are prone to mutations, these regions should be verified using Sanger sequencing using the LKO1-5 primer (5′-GACTATCATATGCTTACCGT-3′).
19. Purify the final plasmid using an appropriate method to achieve transfection-grade purity.

Establishment of STREAMING-tag knock-in cells

Here, we present an example of generating STREAMING-tag knock-in mESCs. When using different cell types, it is necessary to optimize the transfection method and G418 treatment concentration depending on the cell type.

Materials:

- C57BL6J mESC cell line (Bruce 4 C57BL/6J, male, EMD Millipore, Billerica, MA, USA)
- Targeting vector (see above)
- CRISPR vector (see above)
- pKLV-PGKpuro2ABFP (Addgene # 122372)
- 2i medium (Dulbecco's modified Eagle's medium [DMEM]; 15% fetal bovine serum [FBS]; 0.1 mM β-mercaptoethanol; 1× MEM nonessential amino acids; 2 mM L-alanyl-L-glutamine solution; 1,000 U/mL leukemia inhibitory factor [LIF]; 20 μg/mL gentamicin; 3 μM CHIR99021; and 1 μM PD0325901)
- Opti-MEM reduced serum medium (Life Technologies, Carlsbad, CA, USA; 11058021)
- Lipofectamine 3000 (Life Technologies, L3000015)
- Puromycin (Wako, 160-23151)
- G418 (Nacalai Tesque, 16512-81)

Protocols:

1. Day 0
    1. Plate mESCs (5×10^5) into each well of a 12-well plate, and after 1 h, mix the following transfection reagents: 2 μg of targeting vector (e.g., pTV-Nanog_2-5prime-1000-24MS96T-3F_NeoR), 700 ng of CRISPR vector (e.g., eSpCas9-EF-5Nanog_2), and 300 ng of pKLV-PGKpuro2ABFP.
    2. To each of these, add 62.5 μL of reduced serum Opti-MEM and 2.5 μL of P3000 reagent.
    3. In a separate tube, add 62.5 μL of reduced serum Opti-MEM and 4.5 μL of Lipofectamine 3000 per reaction mixture, and mix well.
    4. Mix these reagents in equal volumes and incubate for 15 min at room temperature. Add the complex to wells containing cells, and incubate overnight.
2. Day 1

    Replace the medium with fresh 2i medium containing 1 μg/mL puromycin.

3. Day 2
   Replace the medium with fresh 2i medium.
4. Day 3
   Transfer all cells to gelatin-coated 10 cm dishes.
5. Day 5
   Replace the medium with 8 mL of 2i medium containing 200 μg/mL G418.
6. Day 7
   Replace the medium with 8 mL of 2i medium containing 200 μg/mL G418.
7. Day 9
   After another 48 h, pick up 24 colonies.

Genomic DNA is extracted from a portion of each colony, and knock-in candidate colonies are selected using genomic PCR. In addition, after expanding the candidate colonies, genomic DNA is extracted from a portion of each colony, and the rest is cryopreserved. Knock-in and random integration of vectors are confirmed using Southern blotting.

Establishment of fluorescent protein-expressing cells using the piggyBac system

Here, we present an example of mESCs. When using different cell types, it is necessary to optimize the transfection method and G418 treatment concentration depending on the cell type.

Materials:

- STREAMING-tag knock-in mESC lines
- 2i medium (Dulbecco's modified Eagle's medium [DMEM]; 15% fetal bovine serum [FBS]; 0.1 mM β-mercaptoethanol; 1×MEM nonessential amino acids; 2 mM L-alanyl-L-glutamine solution; 1,000 U/mL leukemia inhibitory factor [LIF]; 20 μg/mL gentamicin; 3 μM CHIR99021; and 1 μM PD0325901)
- pCAG hyPBase[3]
- pLR5-CAG-TetR_W43F-3xmNG (Addgene #174880)
- pLR5-CAG-hMCP-mScarlet-I-NLS (Addgene #174878)
- SNAPtag expression vector (e.g., pLR5-CAG-NLS-SNAP, Addgene #174879)
- Opti-MEM reduced serum medium (Life Technologies, Gaithersburg, MD, USA; 11058021)

- Lipofectamine 3000 (Life Technologies, L3000015)
- SNAP-Cell 647-SiR (New England Biolabs, S9102S)

Protocols:

1. Day 0
    1. Plate STREAMING-tag knock-in mESC lines (2.5×10^5) to each well of a 24-well plate; after 1 h, mix the transfection reagents as described below.
    2. In a tube, mix 50 ng pCAG hyPBase, 75 ng pLR5-CAG-TetR_W43F-3xmNG, 275 ng pLR5-CAG-hMCP-mScarlet-I-NLS, and 100 ng of SNAPtag expression vector (pLR5-CAG-NLS-SNAP).
    3. To each of these, add 25 μL of reduced serum Opti-MEM and 1 μL of P3000 reagent.
    4. In a separate tube, add 25 μL of reduced serum Opti-MEM and 1.8 μL of Lipofectamine 3000 per reaction mixture, and mix well.
    5. Mix these reagents in equal volumes and incubate at room temperature for 15 min.
2. Day 1

    Replace the medium with 2i medium.
3. Day 2

    Replace the medium with 2i medium.
4. Day 5

    Passage the cells to 12-well plates.
5. Day 6
    1. Incubate the cells in 2i medium containing 300 nM SNAP-Cell 647-SiR for 30 min at 37 °C and 5% CO_2 for another 30 min.
    2. Collect the cells by trypsin treatment, and cells moderately expressing mTetR-mNG, MCP-RFP, and SNAPtag should be sorted using a BD FACSAria III cell sorter (BD Biosciences, Franklin Lakes, NJ, USA) and seeded onto gelatin-coated 6 cm dishes. The cells should show a mild level of fluorescence expression; if the expression level is too high, it will be difficult to detect the clusters (Supplementary Fig. 10).
6. Day 8

    Replace the medium with 2i medium, and change the medium once every 2 days.
7. Day 11

Observe cells expressing moderate levels of fluorescent protein under a fluorescence microscope, and use these cells for further experiments.

References

1. Bieberstein, N. I., Carrillo Oesterreich, F., Straube, K. & Neugebauer, K. M. First Exon Length Controls Active Chromatin Signatures and Transcription. *Cell Rep* 2, 62–68 (2012).

2. Tasaki, T., Sriram, S. M., Park, K. S. & Kwon, Y. T. The N-End Rule Pathway. *Annu Rev Biochem* 81, 261–289 (2012).

3. Ochiai, H., Sugawara, T. & Yamamoto, T. Simultaneous live imaging of the transcription and nuclear position of specific genes. *Nucleic Acids Res* 43, e127–e127 (2015).
