# Supplementary material for: STREAMING-tag system reveals spatiotemporal relationships between transcriptional regulatory factors and transcriptional activity
Source: Nat Commun. 2022 Dec 20;13:7672. doi: 10.1038/s41467-022-35286-2 (PMC9768169; doi:10.1038/s41467-022-35286-2)
Supplement: Supplementary file 3 — Description of additional Supplementary File [file 41467_2022_35286_MOESM3_ESM.pdf]

Descriptions of Additional Supplementary Files

Supplementary Data 1: Cell lines used in this study

Supplementary Data 2: Plasmids used in this study

Supplementary Data 3: Southern blot probes and primers used to generate the probes

Supplementary Data 4: Nucleotide sequences of smFISH probes used in this study

Supplementary Movie 1. Dynamics of MS2 coat protein (MCP) and mTetR spots in NStNLS-SNAP cells. NSt-NLS-SNAP cells were treated with SNAP-Cell 647-SiR immediately before imaging. The movie shows a maximum intensity projection-processed timelapse image of NSt-NLS-SNAP cells captured at 15 s intervals. Scale bar, 5 µm.
