# Supplementary material for: STREAMING-tag system reveals spatiotemporal relationships between transcriptional regulatory factors and transcriptional activity
Source: Nat Commun. 2022 Dec 20;13:7672. doi: 10.1038/s41467-022-35286-2 (PMC9768169; doi:10.1038/s41467-022-35286-2)
Supplement: Supplementary file 9 — Reporting Summary [file 41467_2022_35286_MOESM9_ESM.pdf]

nature portfolio

Corresponding author(s): Hiroshi Ochiai, Hiroshi Kimura

Last updated by author(s): Nov 25, 2022

Reporting Summary

Nature Portfolio wishes to improve the reproducibility of the work that we publish. This form provides structure for consistency and transparency in reporting. For further information on Nature Portfolio policies, see our Editorial Policies and the Editorial Policy Checklist.

Statistics

For all statistical analyses, confirm that the following items are present in the figure legend, table legend, main text, or Methods section.

n/a	Confirmed	
☐	☒	The exact sample size (*n*) for each experimental group/condition, given as a discrete number and unit of measurement
☐	☒	A statement on whether measurements were taken from distinct samples or whether the same sample was measured repeatedly
☐	☒	The statistical test(s) used AND whether they are one- or two-sided<br>*Only common tests should be described solely by name; describe more complex techniques in the Methods section.*
☒	☐	A description of all covariates tested
☐	☒	A description of any assumptions or corrections, such as tests of normality and adjustment for multiple comparisons
☐	☒	A full description of the statistical parameters including central tendency (e.g. means) or other basic estimates (e.g. regression coefficient) AND variation (e.g. standard deviation) or associated estimates of uncertainty (e.g. confidence intervals)
☐	☒	For null hypothesis testing, the test statistic (e.g. *F*, *t*, *r*) with confidence intervals, effect sizes, degrees of freedom and *P* value noted<br>*Give P values as exact values whenever suitable.*
☒	☐	For Bayesian analysis, information on the choice of priors and Markov chain Monte Carlo settings
☒	☐	For hierarchical and complex designs, identification of the appropriate level for tests and full reporting of outcomes
☒	☐	Estimates of effect sizes (e.g. Cohen's *d*, Pearson's *r*), indicating how they were calculated

Our web collection on statistics for biologists contains articles on many of the points above.

Software and code

Policy information about availability of computer code

Data collection	NIS-elements (version 5.11.01) was used for imaging. BD FACSDiva Software (version 8.0.1) was used for FACS analysis and cell sorting.
Data analysis	Detection and counting of smFISH signals were performed using Big-FISH (version 0.6.2). Most images were analyzed using a combination of ImageJ (version 2.0.-rc-69/1.52i) and Python (version 3.9.6). Some images were analyzed using Imaris software (version 9.1.2). R (version 4.1.1) and Python (version 3.9.6) were used for graph plotting. Cellpose (version 2.1.0) was used for nucleus segmentation.<br>All codes used in this study have been reported previously and any minor modifications are described. Python code used to measure the distance between the STREAMING-tag region and the RF cluster is available from GitHub under https://github.com/Ochiai-Lab/STREAMING-tag.

For manuscripts utilizing custom algorithms or software that are central to the research but not yet described in published literature, software must be made available to editors and reviewers. We strongly encourage code deposition in a community repository (e.g. GitHub). See the Nature Portfolio guidelines for submitting code & software for further information.

Data

Policy information about availability of data

All manuscripts must include a data availability statement. This statement should provide the following information, where applicable:

- Accession codes, unique identifiers, or web links for publicly available datasets
- A description of any restrictions on data availability
- For clinical datasets or third party data, please ensure that the statement adheres to our policy

Data supporting the findings of this study are available within the article, Supplementary Information and Source Data, and are also available from the corresponding author on reasonable request. Source data underlying Fig. 1-7 as well as Supplementary Fig. 2–10 are provided as a Source Data file.

Field-specific reporting

Please select the one below that is the best fit for your research. If you are not sure, read the appropriate sections before making your selection.

[x] Life sciences [ ] Behavioural & social sciences [ ] Ecological, evolutionary & environmental sciences

For a reference copy of the document with all sections, see nature.com/documents/nr-reporting-summary-flat.pdf

Life sciences study design

All studies must disclose on these points even when the disclosure is negative.

Sample size	No statistical methods were used to pre-determine sample sizes, which were chosen based on previous experience with these metrics. The exact number, n, of data points and their representation (such as cells and independent experiments), and statistical tests used are indicated in the respective figure legends and in the results.
Data exclusions	No data were excluded.
Replication	All experiments were performed as two or more independent experiments. The same conclusions were obtained from each experiment.
Randomization	Randomization is not relevant to our study as the analysis of microscopy data was automated in an unbiased manner. Parameters, such as thresholding for spot detection, were consistent in each experiment.
Blinding	Blinding was not relevant for our study since our analyses were analyst-independent.

Reporting for specific materials, systems and methods

We require information from authors about some types of materials, experimental systems and methods used in many studies. Here, indicate whether each material, system or method listed is relevant to your study. If you are not sure if a list item applies to your research, read the appropriate section before selecting a response.

Materials & experimental systems

n/a	Involved in the study	
[ ]	[x]	Antibodies
[ ]	[x]	Eukaryotic cell lines
[x]	[ ]	Palaeontology and archaeology
[x]	[ ]	Animals and other organisms
[x]	[ ]	Human research participants
[x]	[ ]	Clinical data
[x]	[ ]	Dual use research of concern

Methods

n/a	Involved in the study	
[x]	[ ]	ChIP-seq
[ ]	[x]	Flow cytometry
[x]	[ ]	MRI-based neuroimaging

Antibodies

Antibodies used	anti-mNeonGreen [Chrom Tech, Cat#32f6-100; RRID: AB_2827566]<br>GAPDH XP Rabbit mAb antibody [Cell Signaling, Cat#5174; RRID: AB_10622025]<br>Nanog monoclonal antibody [eBioscience, Cat#14-5761-80; clone nema : eBioMLC-51; RRID: AB_763613]<br>Anti-SOX2 antibody [Abcam, Cat#ab97959; RRID: AB_2341193]<br>USP5 antibody [Proteintech, Cat#10473-1-AP; RRID: AB_2272754]<br>Mouse IgG for Ser2ph [In-house 54, CMA602; RRID: AB_2819246]<br>Mouse IgG for Ser5ph [In-house 54, CMA603; RRID: AB_2827955]<br>Anti-GFP HRP-DirecT antibody [MBL, Cat# 598-7; RRID:AB_10597267]<br>Peroxidase-AffiniPure Goat Anti-Mouse IgG (H + L) antibody [Jackson ImmunoResearch, Cat#115-035-003; RRID:AB_10015289]<br>mouse anti-RPB1 antibody [CMA601, in house]
Validation	All antibodies used in this research were purchased commercially or previously published (ref 57). All primary antibodies have been validated by the manufacturers or the original developer (ref 57).

Eukaryotic cell lines

Policy information about cell lines

Cell line source(s)	- Mouse Bruce4 C57BL/6J (male) embryonic stem cell line (EMD Millipore) and KI derivatives<br>- Mouse C57BL/6NCr (male) embryonic stem cell line established by Tadashi Okamura in National Center for Global Health and Medicine (NCGM) (Ochiai et al., Sci Adv, 2020)

	- HeLa cells (CCL-2, ATCC)
Authentication	All mouse ES cell lines were confirmed to express pluripotent markers by immunofluorescence. Karyotypes of mouse Bruce4 and C57BL/6NCr embryonic stem cell lines were normal. Mouse Bruce4 and C57BL/6NCr knock-in derivatives were analyzed by Southern blot analysis.
Mycoplasma contamination	All cell lines were tested negative for mycoplasma contamination
Commonly misidentified lines (See ICLAC register)	No commonly misidentified cell lines were used.

Flow Cytometry

Plots

Confirm that:

☒ The axis labels state the marker and fluorochrome used (e.g. CD4-FITC).

☒ The axis scales are clearly visible. Include numbers along axes only for bottom left plot of group (a 'group' is an analysis of identical markers).

☒ All plots are contour plots with outliers or pseudocolor plots.

☒ A numerical value for number of cells or percentage (with statistics) is provided.

Methodology

Sample preparation	Cells were washed with PBS, trypsinized, inactivated with FluoroBrite DMEM containing 10% FBS, and centrifuged to collect the cells. Cells were suspended in PBS to be 1 to 5 × 10^6 cells/mL.
Instrument	BD FACSAria III
Software	BD FACSDiva Software version 8.0.1
Cell population abundance	Clonal cell lines were used for all experiments. Most of the cells (around 70%) were within the post-sort fractions in the preliminary FSC-SSC gating.
Gating strategy	The gating strategy was initially FSC-A by SSC-A, followed by doublet exclusion using FSC-H and SSC-H.

☒ Tick this box to confirm that a figure exemplifying the gating strategy is provided in the Supplementary Information.
